# Peruvoside, a Cardiac Glycoside, Induces Primitive Myeloid Leukemia Cell Death

**DOI:** 10.3390/molecules21040534

**Published:** 2016-04-22

**Authors:** Qian Feng, Wa Seng Leong, Liang Liu, Wai-In Chan

**Affiliations:** State Key Laboratory of Quality Research in Chinese Medicine, Macau University of Science and Technology, Macau, China; fengqianmoon@163.com (Q.F.); michael_jackydog@hotmail.com (W.S.L.)

**Keywords:** cardiac glycoside, Peruvoside, acute myeloid leukemia

## Abstract

Despite the available chemotherapy and treatment, leukemia remains a difficult disease to cure due to frequent relapses after treatment. Among the heterogeneous leukemic cells, a rare population referred as the leukemic stem cell (LSC), is thought to be responsible for relapses and drug resistance. Cardiac glycosides (CGs) have been used in treating heart failure despite its toxicity. Recently, increasing evidence has demonstrated its new usage as a potential anti-cancer drug. Ouabain, one of the CGs, specifically targeted CD34^+^CD38^−^ leukemic stem-like cells, but not the more mature CD34^+^CD38^+^ leukemic cells, making this type of compounds a potential treatment for leukemia. In search of other potential anti-leukemia CGs, we found that Peruvoside, a less studied CG, is more effective than Ouabain and Digitoxin at inducing cell death in primitive myeloid leukemia cells without obvious cytotoxicity on normal blood cells. Similar to Ouabain and Digitoxin, Peruvoside also caused cell cycle arrest at G_2_/M stage. It up-regulates CDKN1A expression and activated the cleavage of Caspase 3, 8 and PARP, resulting in apoptosis. Thus, Peruvoside showed potent anti-leukemia effect, which may serve as a new anti-leukemia agent in the future.

## 1. Introduction

Acute myeloid leukemia (AML) is characterized by overproduction of immature white blood cells. Despite available therapies, leukemia remains a difficult disease to cure as relapse often happens post treatment. Evidence has demonstrated that genetic lesions occur in the hematopoietic stem or progenitor cells, resulting in differentiation block due to acquisition of abnormal self-renewal program [1,2]. These leukemia-initiating cells or leukemic stem cells (LSCs) are believed to be the cause of disease relapse as they are often resistant to current therapy due to their stem cell-like properties [3]. The development of more effective new treatments that target these properties and cure leukemia is still much needed.

Natural product remains the main source of drug development. For example, Parthenolide, a component of the medicinal plant feverfew, has demonstrated its apoptotic effect on LSCs as well as bulk leukemic cells [4,5]. CGs are derived from plants and have been used to treat cardiac failure and chronic heart failure [6,7]. Recently, more and more evidence has demonstrated its apoptotic effect in many different types of cancers, including colon cancer, lung cancer, breast cancer and leukemia [8,9,10,11,12], suggesting its potential new application as an anti-cancer drug. Ouabain and Digitoxin are two members of CGs commonly used in clinic for treatment of cardiac conditions. Recently, these two CGs were shown to be effective in targeting human T and B cell leukemia, as well as AML [13,14] at a concentration lower than that used in cardiac treatment. Interestingly, Ouabain was shown to be more effective at targeting the CD34^+^CD38^−^ leukemic stem cell-like population than the CD34^+^CD38^+^ leukemia cells [14], which makes this type of compounds an interesting potential anti-LSCs drug candidate.

It is well known that CGs act on cardiac tissues by inhibiting Na^+^/K^+^-ATPase, resulting in increase of intracellular sodium ions [15]. However, the anti-cancer mechanisms of CGs are still not clear. The Na^+^/K^+^ pump not only functions as a membrane transporter, it is also involved in various signal transductions and cellular processes relating to cell death and survival, *etc.* [10,16]. CGs may act on Na^+^/K^+^-ATPase in a way different from its standard role in normal homeostasis. In fact, it was shown that, Digitoxin, when used at nanomolar concentrations, triggered multiple signal transductions via the Na^+^/K^+^ pump resulting in anti-cancer effects in pancreatic cancer cells [9,17]. Studies have also suggested that different types of cancer cells showed various sensitivities to different CGs, possibly due to different cellular content [8,9,18].

Peruvoside, a less studied CG, is naturally derived from *Thevetia neriifolia* [19]. Its cardiac effect was higher than Ouabain, Digoxin and other commonly used CGs with a therapeutic window wider than other CGs [20]. Peruvoside also demonstrates anti-cancer activities. Li H *et al.* reported that Peruvoside inhibited cell growth in androgen-resistant LNCaP-abl prostate at 50 nM, lower than the concentrations required by both Digoxin and Strophanthidin (500 nM) [21], suggesting that it may be a more potent anti-cancer candidate that other CGs. Here we report that Peruvoside induced apoptotic cell death in human primitive AML KG1a cells and chronic myelogenous leukemia (CML) cell K562. Peruvoside treatment of these cells resulted in more apoptotic cells as compared to Digitoxin and Ouabain. Importantly, it did not show obvious cytotoxicity on normal human peripheral blood mononuclear cells (PBMCs) at the effective dose, demonstrating that Peruvoside could act as a potential anti-leukemia agent. Similar to Ouabain and Digitoxin, Peruvoside also arrested leukemic cells at G_2_/M phase and activated the cleavage of Caspase 8 and Caspase 3 apoptotic pathway in KG1a cells but not in K562 cells, indicating that despite different potency of individual CG, they may trigger cell death by targeting a similar apoptotic pathway.

## 2. Results

### 2.1. Cytotoxicity of Peruvoside on Primitive Leukemia Cells

**Figure 1:**
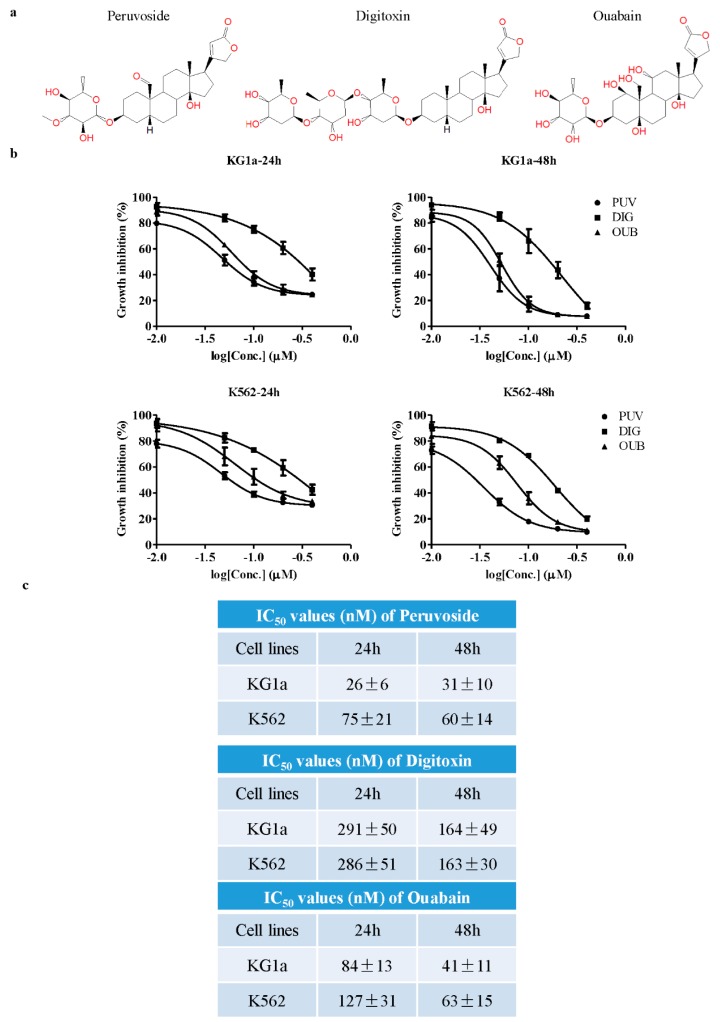
Peruvoside is more effective in suppressing the growth of human leukemia cells. (**a**) Structure of Peruvoside, Digitoxin and Ouabain; (**b**) Dose response of three different cardiac glycosides (CGs) in human leukemia cell line KG1a and K562 at two different time points. MTT assay was used to assess cell viability 24 and 48 h after treatment (*n* = 3); (**c**) IC_50_ values of different CGs 24 and 48 h after treatment (*n* = 3).

We first tested the cytotoxicity effects of Peruvoside on K562 and KG1a cells. Ouabain and Digitoxin were included as comparison. Their structures are shown in Figure 1a. K562 represents CML blast crisis, consisting of primitive blast-like leukemic cells, while the CD34^+^CD38^−^ population of KG1a demonstrated the leukemia stem-like cell property *in vivo* [22]. Therefore they represented two initial cell models to test the potential of candidate compounds in targeting the leukemia blast or stem-like cells. As shown in Figure 1b,c, the IC_50_ values in KG1a at 24 and 48 h were 26 ± 6 nM and 31 ± 10 nM as compared to 75 ± 21 nM and 60 ± 14 nM in K562, respectively, suggesting that KG1a was more sensitive to Peruvoside than K562. However, the IC_50_ values of Digitoxin in both KG1a and K562 were similar, while the IC_50_ of Ouabain was slightly lower in KG1a than in K562. Compared to Digitoxin and Ouabain, Peruvoside was more potent in suppressing the growth of these two leukemic cells. The IC_50_ values of Peruvoside in KG1a and K562 cells were the lowest among all three CGs at 24 h. However, at 48 h, while there were obvious differences in IC_50_ values between Peruvoside and Digitoxin, Peruvoside shared similar IC_50_ to that of Ouabain. We also observed obvious proliferation inhibition after Peruvoside treatment under the microscope. These data demonstrated that Peruvoside is more effective at suppressing cell growth in leukemia than the more commonly used CGs Ouabain and Digitoxin, although at the later time point, the effects of Ouabain and Peruvoside may become similar as suggested by their IC_50_ values. Furthermore, the primitive AML cell KG1a is more sensitive to Peruvoside than the CML blast cell K562.

### 2.2. Peruvoside Induces Apoptosis in Human Leukemia Cells

**Figure 2:**
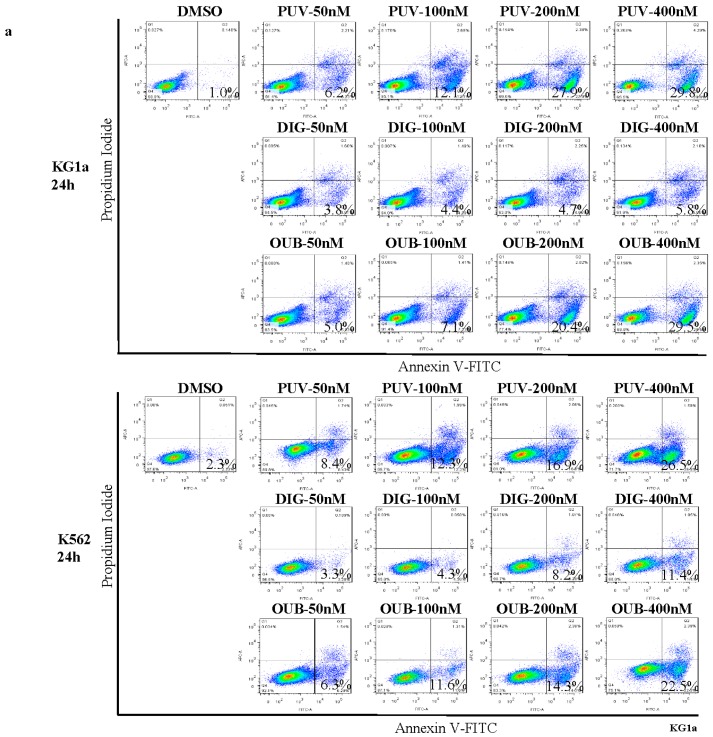

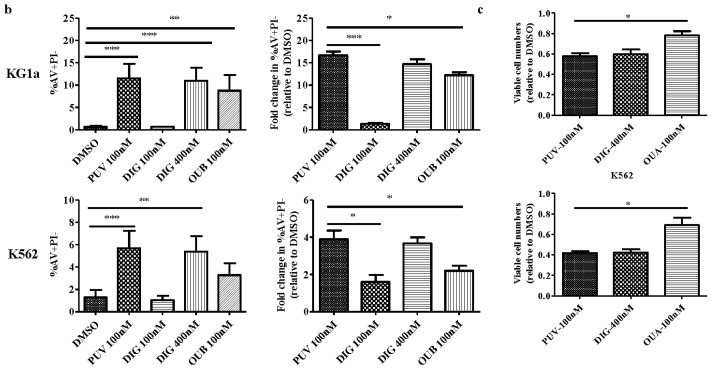
Peruvoside is more effective in inducing apoptosis in human leukemia cells. (**a**) Flow cytometry analysis of Annexin V (AV) and Propidium Iodide (PI) expression in KG1a and K562 treated with various CGs at different concentrations for 24 h; (**b**) Graphs show the percentage of early apoptotic cells (AV^+^PI^−^) measured in (**a**) as well as the fold changes in the percentage of AV^+^PI^−^ (treatment/DMSO); (**c**) Graphs show the fold changes in viable cell numbers (treatment/DMSO). PUV, Peruvoside; DIG, Digitoxin; OUB, Ouabain. *n* ≥ 3, * *p* < 0.05; ** *p* < 0.01, *** *p* < 0.0001.

We next tested if the growth inhibition we detected was due to induction of apoptosis by flow cytometry analysis of Annexin V (AV) and Propidium Iodide (PI) positivity 24 h after treatment (Figure 2a,b). All CGs induced apoptosis in a dose–dependent manner. Compared to Ouabain and Digitoxin, treatment with Peruvoside at different concentrations induced the highest percentage of apoptosis events in both KG1a and K562, although the difference between Peruvoside and Ouabain at 400 nM in KG1a was no longer obvious. The percentages of early apoptotic cells treated with Peruvoside at 100 nM increased by more than 15 fold in KG1a and 4 fold in K562, as compared to those treated with DMSO (Figure 2a,b). However, Digitoxin at the same concentration did not cause obvious apoptosis in both cell lines as the percentages of AV^+^PI^−^ were similar to that of DMSO. Only when its concentration was increased to 400 nM, the percentages of early apoptotic cells were comparable to those treated with Peruvoside. When comparing the fold change in AV^+^PI^−^ cells over DMSO, Peruvoside treatment showed the largest change among three CGs at the same concentration in both cell lines, which was more obvious in KG1a than in K562. The absolute viable cell counts on treated cells using the trypan blue exclusion method also confirmed that there were relatively less viable cells in Peruvoside treated group than in Ouabain treated group at the same concentration in both cell lines (Figure 2c). The differences were mild but statistically significant. These data demonstrated that Peruvoside is more effective than other two anti-leukemia CGs, Ouabain and Digitoxin, in inducing apoptosis in human leukemic cells.

### 2.3. Peruvoside Does Not Show Obvious Cytotoxicity on Normal Blood Cells

**Figure 3:**
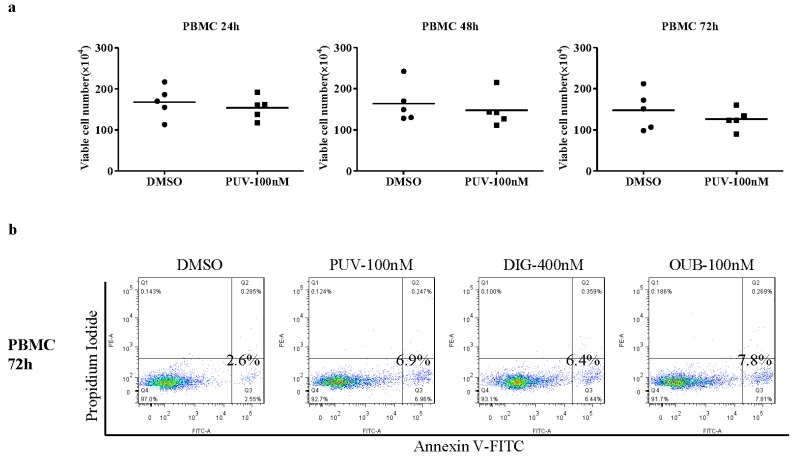
Peruvoside did not show obvious cytotoxicity on normal human peripheral blood mononuclear cells (PBMCs). (**a**) Viable cell numbers at different time points after Peruvoside treatment; (**b**) Flow cytometry analysis of Annexin V and PI expression in PBMCs after being treated with various CGs for 72 h. PUV, Peruvoside; DIG, Digitoxin; OUB, Ouabain.

CGs have been shown to be toxic to most cell types, which hampers their clinical use. As shown above, primitive leukemic cells were sensitive to Peruvoside. Therefore, Peruvoside at its effective dose may not cause significant cytotoxicity on normal cells. To investigate its cytotoxicity effect on normal blood cells, we treated normal peripheral blood mononuclear cells (PBMCs) with Peruvoside at its effective dose of 100 nM. As shown in Figure 3a, Peruvoside treatment at 100 nM did not cause obvious cell death after 24, 48 and 72 h of treatment as shown by the total numbers of live cells. Annexin V and PI staining on cells treated with CGs for 72 h showed that Peruvoside at 100 nM caused a mild increase in the percentage of AV^+^PI^−^ population, around 2.7 fold over DMSO, which was similar to that of Digitoxin at 400 nM (2.5 fold over DMSO) and Ouabain 100 nM (3 fold over DMSO) (Figure 3b). This increase, compared to an increase of around 15 fold and 4 fold in KG1a and K562, respectively (Figure 2b), was mild, suggesting a possible therapeutic window for Peruvoside in treating leukemia.

### 2.4. Peruvoside Induces G_2_/M Phase Arrest in KG1a and K562 Cells

**Figure 4:**
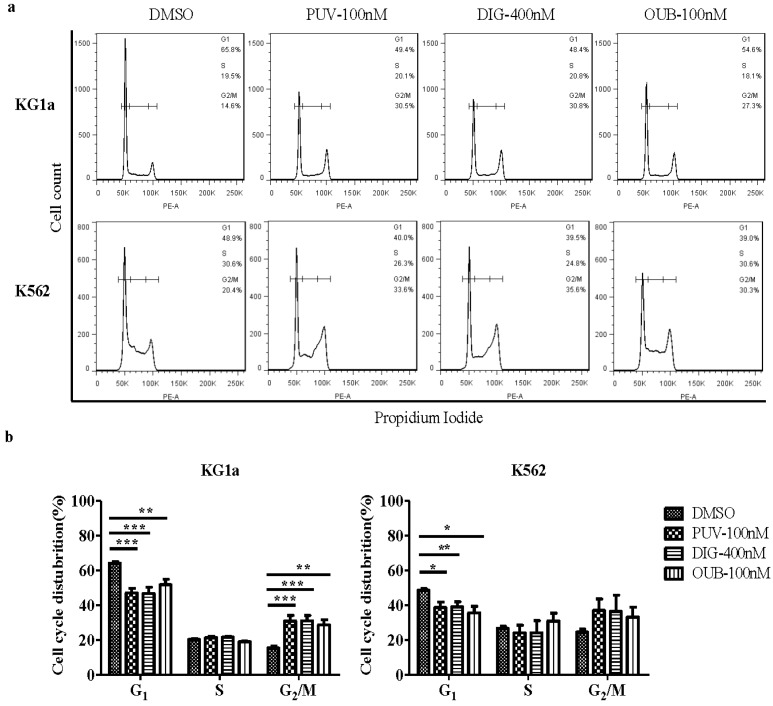
Peruvoside induces cell cycle arrest at G_2_/M, at a level similar to Digitoxin and Ouabain. (**a**) Cells treated with Peruvoside (100 nM), Digitoxin (400 nM) and Ouabain (100 nM) were stained with PI. Changes in cell cycle distribution of treated cells were analyzed by flow cytometry; (**b**) Graphs show cell cycle distributions of cells treated with different CGs shown in (**a**); PUV, Peruvoside; DIG, Digitoxin; OUB, Ouabain. *n* = 3, *, *p* < 0.05; **, *p* < 0.01, ***, *p* < 0.0001.

To investigate whether Peruvoside also induced cell cycle arrest, we exposed KG1a and K562 cells to 100 nM of Peruvoside for 24 h. As shown in Figure 4, Peruvoside significantly increased the percentage of KG1a cells in G_2_/M phase and decreased the numbers of KG1a cells in G_1_ phase, to a level similar to those treated with 400 nM of Digitoxin and 100 nM of Ouabain. However, in K562, treatment with all CGs tested only decreased the percentages of G_1_ phase cells, with a trend of increase in the numbers of G_2_/M cells. Nevertheless, these data, in agreement with the reported effects of CGs on G_2_/M accumulation [16], suggests that arresting cancer cells at G_2_/M stage may be a common effect of CGs.

### 2.5. Peruvoside Does Not Alter the Intracellular Reactive Oxygen Species (ROS) Level in Leukemia Cells

**Figure 5:**
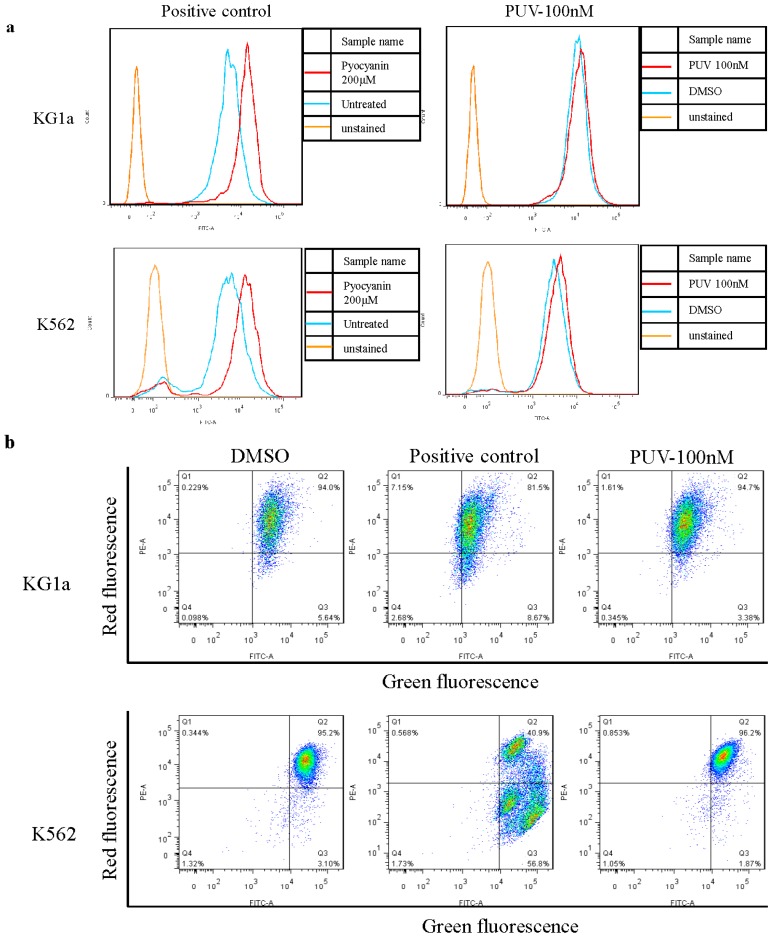
Peruvoside treatment did not alter the intracellular Reactive Oxygen Species (ROS) level nor the loss of MMR in KG1a and K562 cells. (**a**) Both cell lines were treated with 100 nM of Peruvoside or 200 nM of Pyocyanin for 2 h before stained with ROS indicator; (**b**) Both cell lines were treated with a positive control or 100 nM of Peruvoside for 24 h followed by JC-1 staining. Fluorescence signal was measured and analyzed by flow cytometry. PUV: Peruvoside.

Intracellular ROS plays an important role in inducing apoptosis in cancer cells. Previous study showed that Ouabain elevated the cytosolic ROS level in U937 cells [23] and neuroblastoma cell line SH-SY5Y cells [24]. In order to investigate if Peruvoside also induced ROS production in KG1a and K562 leading to apoptosis, we assessed the ROS level in these cells by flow cytometry 24 h after Peruvoside treatment. As shown in Figure 5a, treatment of Peruvoside at 100 nM did not lead to a significant change in ROS level in both KG1a and K562 cells, while the positive control (200 nM of Pyocyanin) induced an obvious increase in ROS level in these cells. This data suggested that apoptosis induced by Peruvoside treatment was not due to increased intracellular ROS level.

### 2.6. Peruvoside Does Not Affect the Mitochondrial Membrane Potential (MMP) in Leukemia Cells

It has been reported that Ouabain triggered apoptosis by interfering the mitochondrial function in androgen-independent prostate cancer cells [25]. To examine if Peruvoside treatment also results in a change in MMP, cells treated with Peruvoside for 24 h were stained with the fluorescence dye JC-1 followed by flow cytometry analysis. Changes of fluorescence in treated cells from red (high MMR) to green (low MMT) indicate depolarization of mitochondria followed by cell undergoing apoptosis. However, unlike the positive control, Peruvoside did not alter the green fluorescence signal level in both KG1a and K562 cells (Figure 5b), indicating that 24 h of Peruvoside treatment did not affect the MMP in both cell lines.

### 2.7. Peruvoside Induces Up-Regulation of Cyclin-Dependent Kinase Inhibitor 1A

**Figure 6:**
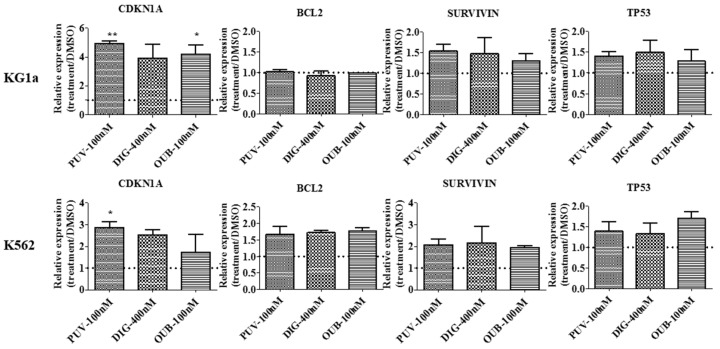
Up-regulation of cyclin-dependent kinase inhibitor 1A in leukemia cells after Peruvoside treatment. KG1a and K562 cells were treated with different CGs for 24 h. RNA was isolated from each sample and cDNA was synthesized for Real-time PCR analyses with gene specific primers using the SYBR green method. Data was normalized to β-actin expression and the relative quantity of gene expression level was calculated by ΔΔCt method. Changes in expression are presented as fold change relative to DMSO. Dot line indicates DMSO value set as 1. PUV, Peruvoside; DIG, Digitoxin; OUB, Ouabain. *n* = 3, *, *p* < 0.05; **, *p* < 0.01.

To compare if Peruvoside caused similar effects as Digitoxin and Ouabain at the molecular level, we examined the expression level of several genes involved in cell survival and cell cycle regulation using real-time PCR analysis. As shown in Figure 6, Peruvoside treatment resulted in up-regulation of Cyclin-Dependent Kinase Inhibitor 1A (CDKN1A) expression in KG1a up to 6 fold, highest compared to Digitoxin treatment at 400 nM and Ouabain at 100 nM. In K562, Peruvoside also induced the highest level of CDKN1A expression among other CGs tested. CDKN1A is an important regulator during G_1_ and G_2_/M phase progression. Up-regulation of this gene is; therefore, in agree with the effect of CGs on cell cycle in KG1a and K562 observed in our study. CDKN1A expression is also up-regulated in response to DNA damage via p53 activation. However, TP53 RNA level was not changed in both cell lines after different CGs treatment. Expression of genes related to cell survival, such as SURVIVIN and BCL2 were also not affected by CGs treatment.

### 2.8. Peruvoside Activates the Cleavage of Caspase Proteins in Leukemia Cells

**Figure 7:**
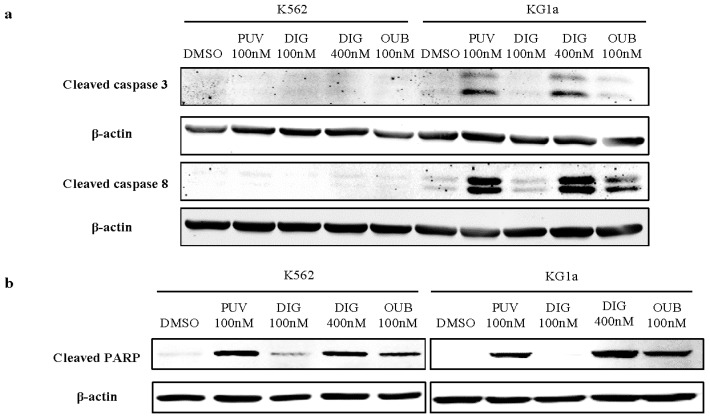
Peruvoside activates the apoptosis associated Caspase pathway. Western-blotting analysis shows that among other CGs, treatment of Peruvoside (PUV) at 100 nM for 24 h induced the highest level of cleaved Caspase 3 and 8 expression in KG1a. Treatment with Digitoxin (DIG) at 400 nM induced similar level of Caspase 3 and 8 expression as Peruvoside at 100 nM. Peruvoside also induced the expression of cleaved PARP in both K562 and KG1a. PUV, Peruvoside; DIG, Digitoxin; OUB, Ouabain.

We next examined the Caspase activity in KG1a and K562 treated with CGs or DMSO (Figure 7). Western blotting demonstrated the induction of cleaved PARP in KG1a cells at a level similar to Digitoxin at 400 nM and Ouabain at 100 nM. When cells were treated with these CGs at the same concentration (100 nM), Peruvoside induced the highest level of cleaved Caspase 3 and 8 compared to Digitoxin and Ouabain (Figure 7). Interestingly, in K562, treatments of all three CGs did not activate the cleavage of Caspase-3 and 8 but resulted in cleaved PARP expression, suggesting that CGs may target different signaling components in AML and CML cells resulting in PARP cleavagee. These data indicated that, similar to Ouabain and Digitoxin, Peruvoside induced apoptosis via the classical Caspase associated apoptotic pathway in both KG1a and K562 cells.

## 3. Discussion

Increasing amounts of studies have discovered new clinical applications for old drugs. For example, Metformin, the first line treatment for diabetes, has been demonstrated to exhibit anti-cancer properties to a range of cancers [26]. Similarly, CGs have a long history in treating heart failure in clinical situations. More and more evidences have suggested its new use as potential anti-cancer agent. The treatment window of CGs may be small; however, due to higher sensitivity of cancer cells to CGs treatment, highly potent CGs may still be able to target certain types of cancer within a safety range [27]. Among commonly used CGs, Ouabain and Digitoxin were shown to be effective in targeting human leukemia. Here we demonstrated that Peruvoside, a less studied CG, is more effective than Ouabain and Digitoxin in causing cell death in human primitive AML cells KG1a and CML cells K562. It showed relatively low IC_50_ values in both cell lines, and induced the highest degree of apoptosis compared to Digitoxin and Ouabain. Our data is consistent with a previous study on pancreatic cancer, showing that the cytotoxic potency of Peruvoside is the highest among nine cardiac glycosides against six pancreatic cancer cell lines [17]. This study, together with our present data, indicates that Peruvoside is a more effective anti-cancer compound than other CGs. Moreover, we have shown that Peruvoside did not cause obvious cytotoxicity on normal blood cells even after 72 h of treatment at the dose that caused cell death in both K562 and KG1a leukemic cells. Apoptosis analysis at this time point showed that Peruvoside as well as two other CGs induced a mild increase in the percentage of early apoptotic cells. Considering the well-known cytotoxicity of CGs and long incubation time (72 h), this result is perhaps expected. However, compared to the percentages of apoptosis induced in leukemia cells (Figure 2), the apoptosis events detected in normal cells were relatively small, implying a possible therapeutic window for Peruvoside in leukemia treatment. *In vivo* study is needed to investigate its toxicity and efficacy in details.

Recent study has shown that Ouabain targeted the CD34^+^CD38^−^ population in human leukemia samples [14]. This population contains cells with leukemic stem cell properties. Although the molecular targets of Ouabain in this population are unclear, this result implies that Ouabain might be a potential anti-LSCs agent. It is tempting to speculate that Peruvoside or other CGs that are structurally similar to Ouabain might also target this population. Further detailed analyses of CGs effects on human leukemia samples with more defined LSC markers would be of much interest.

The cellular and molecular mechanisms underlying the anti-cancer effects of CGs are complex [8]. It is well accepted that CGs exert its cardiac effect via binding to Na^+^, K^+^-ATPase, therefore alternating the intercellular Na^+^, K^+^ levels and stimulating the Ca^2+^ influx. Early studies have also demonstrated that this Na-K-ATPase is also part of signalsome transmitting signals to different downstream mediators depending on different cellular content [28]. Differences in expression of sodium pump subunits between cancer and normal cells may account for different effects of CGs in these cells [29,30]. Moreover, individual CG perhaps exerts its effects via different mechanisms. In this study, we have shown that apoptosis in leukemia cells induced by Peruvoside was not due to an increase of ROS level or loss of mitochondrial membrane potential. Cell cycle arrest at G_2_/M observed in this study may serve as one of the causes of apoptosis induced by Peruvoside. Our data has also shown that Peruvoside, as well as Digitoxin and Ouabain, triggered the cleavage of Caspase 3, 8 and PARP in KG1a cells. The effect of Peruvoside on the expression of these proteins was the highest among the other two CGs, suggesting that these compounds trigger a similar apoptotic program in KG1a leukemia cells. Peruvoside also caused up-regulation of CDKN1A mRNA level but did not cause any changes in other pro-survival gene expression like SURVIVIN and BCL2. On the other hand, all CGs induced cleavage of PARP but not Caspase 3 and 8 in K562 CML cells. CGs may target different signaling pathway components that control the survival of these cells, leading to apoptotic cell death. Further detailed studies such as genome-wide gene expression analysis or proteomics analysis are needed to explore the molecular mechanisms underneath the apoptotic effect of Peruvoside in leukemia.

## 4. Materials and Methods

### 4.1. Chemicals

Peruvoside, Digitoxin and Ouabain were purchased from MicroSource Discovery Stystems, Inc. (Gaylordsville, CT, USA). All compounds were dissolved in DMSO and further diluted in culture medium. The final concentration of DMSO was less than 0.1%.

### 4.2. Cell Culture

Human chronic myelogenous leukemia (CML) K562 cell line and human acute myelogenous leukemia (AML) KG1a cell line were purchased from American Type Culture Collection (ATCC, Manassas, VA, USA). K562 was cultured in Iscove’s Modified Dulbecco’s Medium (IMDM, Thermo Fisher Scientific, Waltham, MA, USA) with 10% bovine fetal serum (FBS, GIBCO), while KG1a was cultured in IMDM containing 20% bovine fetal serum. Healthy blood samples were kindly donated as buffy coats by Macau Red Cross (Macau). Peripheral blood mononuclear cells (PBMCs) were separated using the Ficoll-Paque (GE Healthcare Life Sciences, Waukesha, WI, USA) method according to the manufacturer’s instructions. PBMCs were cultured in RPMI-1640 with 10% FBS 24 h before treatment. All cells were maintained at 37 °C, 5% CO_2_.

### 4.3. MTT Cell Viability Assay

Cells were seeded in 96 well plates at a concentration of 1.5 × 10^4^ cells per well for K562 cells and 4 × 10^4^ cells per well for KG1a cells. 2 h later, cells were treated with Peruvoside, Digitoxin or Ouabain at a series of concentrations. DMSO was used as vehicle control. After 24 h or 48 h treatment, 10 μL of 5 mg/mL 3-(4,5-dimetylthiazol-2-yl)-2, 5-diphenyl-tetrazolium (MTT, Sigma-Aldrich, St. Louis, MO, USA) was added to each well and incubated for at least 2 h at 37 °C. After the MTT crystal had formed, 10% SDS with 5 M HCl was added to each well. Absorbances at 570 nm and 650 nm were measured in a microplate reader (Tecan, Switzerland). The average absorbance value from 4 replicates of each treatment was used to determinate cell viability (CGs treated/DMSO control). Each experiment was performed 3 times with 4 replicate wells per treatment.

### 4.4. Apoptosis and Cell Cycle Analyses

1 × 10^5^ cells were seeded in each well of 24-well plates. After being treated with CGs, cells were harvested and washed with cold PBS with 1% FBS, before processed to Annexin V-FITC and PI staining (Annexin V: FITC Apoptosis Detection Kit, BD Biosciences) according to the manufacturer’s instructions. Fluorescence signal was detected in BD FACS Aria III flow cytometry (BD Biosciences). For cell cycle analysis, treated cells were first fixed in 70% ethanol overnight at −40 °C. Subsequently, cells were washed with PBS with 1% FBS and stained with 50 μg/mL of PI solution containing 0.2 mg/mL of RNase A (Sigma-Aldrich). DNA content was determined by flow cytometry. All data was analyzed using FlowJo software.

### 4.5. Reactive Oxygen Species Analysis

Change in ROS level was detected using the Total ROS/Superoxide Detection Kit (ENZO Life Sciences, Farmingdale, NY, USA). Approximately 1 × 10^5^ of treated cells were washed with 1 × wash buffer, re-suspended in 500 µL of Oxidative Stress Detection Reagent and incubated for 30 min at 37 °C in dark. Cells were then washed with 1 × wash buffer and treated with compounds for different lengths of time. All treatments were performed under normal tissue culture conditions. After incubation, cells were washed with 1 × wash buffer once and re-suspended in 500 µL of 1 × wash buffer before being analyzed by flow cytometry. Pyocyanin-treated cells were used as positive control cells.

### 4.6. Mitochondrial Membrane Potential Analysis

To perform the assay, around 5 × 10^5^ treated cells were stained with 1 μM of Tetraethylbenzimidazolylcarbocyanine iodide (JC-1, Abcam Incorporated) in PBS for 30 min at 37 °C in dark. Afterwards, stained cells were washed twice with PBS and re-suspended in 300 µL of PBS. Each sample was filtered through a filter cap and the fluorescence signal was analyzed by flow cytometry.

### 4.7. Western Blot Analysis

Cells were collected and lysed for 15 min on ice in 1 × RIPA buffer (Cell Signaling Technology, Beverly, MA, USA) containing protease inhibitors (Roche, Lewes, Sussex, UK). The concentration of total protein extract was determined by DCTM protein assay kit (Bio-Rad, Richmond, CA, USA). 40 μg of total protein lysate were loaded onto and electrophoresed in a 10% SDS-PAGE gel and subsequently transferred to PVDF membrane (Roche). The membrane was blocked with 5% skimmed milk in TBST (150 mM NaCl, 10 mM Tris, 0.1% Tween 20, pH 8.0) for 1 h at room temperature before incubated with primary antibodies overnight at 4 °C, followed by secondary antibody incubation at room temperature for 1 h. Primary antibodies used in this study are: Cleaved Caspase 3,6,8,9 and Cleaved PRAP (1:1000, Cell Signaling Technology), β-actin (1:10,000, Sigma). Secondary fluorescent antibodies are: Anti-rabbit IgG Dylight^®^ 680 conjugate, Anti-mouse IgG Dylight^®^ 800 conjugate (1:20,000, Cell Signaling Technology). Fluorescence signal was detected and processed in the ODYSSEY^®^ CLx Infrared Imaging system (LI-COR, Lincoln, NE, USA).

### 4.8. Real-Time Polymerase Chain Reaction Analysis

Total RNA was extracted using the TRIzol^®^ reagent (Thermo Fisher Scientific, Waltham, MA, USA) according to the manufacturer’s instructions. 1 μg of total RNA pre-treated with DNase I (Life Technologies) was used for reverse transcription. cDNA was synthesized using Transcriptor Universal cDNA Master kit (Roche). Real-time quantitative PCR was performed using FS Universal SYBR Green Master (Roche) and signal was detected in the ViiA™ 7 Real-Time PCR System (Life Technologies). RNA expression levels were normalized to that of β-actin, and calculated using the ΔΔCt method. Sequences of the gene specific primers used in this study are available upon request.

### 4.9. Statistical Analysis

Statistical analyses were performed using Prism 6 software (GraphPad, La Jolla, CA, USA). Differences between groups were determined by Student’s *t*-test or one-way analysis of variance (ANOVA), followed by Bonferroni’s test. Data were expressed as mean ± S.E.M from at least three individual experiments.

## 5. Conclusions

Our data suggests that Peruvoside is a new anti-leukemia agent, which is more effective than Ouabain and Digitoxin in inducing apoptotic cell death in human AML and CML cells, via activation of the Caspase pathway. Its relatively low effective dose may overcome the toxicity problem that restricts its clinical use. Further study of its anti-cancer effects *in vivo* will be needed to explore its potential as anti-leukemia therapy.

## Abbreviations

LSC: Leukemic stem cell
CGs: Cardiac glycosides
AML: Acute myeloid leukemia
CML: Chronic myelogenous leukemia
PBMCs: Peripheral blood mononuclear cells
ROS: Reactive oxygen species
MMP: Mitochondrial membrane potential
PUV: Peruvoside
DIG: Digitoxin
OUB: Ouabain
